# Supplementary material for: Morphological and hormonal diversity in rose (Rosa hybrida L.) and potato (Solanum tuberosum L.) Ri genotypes: A comparative study
Source: PLoS One. 2026 Apr 15;21(4):e0345941. doi: 10.1371/journal.pone.0345941 (PMC13082669; doi:10.1371/journal.pone.0345941)
Supplement: S3 File — Supplementary Material and Methods. Details about transformation and regeneration of Ri genotypes, overview of experiment chronology, Material and Methods of dry mass, root hair and plant hormone analysis, and primer overview. (DOCX) [file pone.0345941.s003.docx]

Supplementary Material

Transformation and regeneration

**Am Ri genotypes:**

Am in vitro shoots grown on propagation medium for three weeks were cut freshly at the base and a few millimeters above the base along the stem with a scalpel. The wounded shoots were then transferred into a bacterial solution with the *R. rhizogenes* strain ATCC 15834, which was prepared according to Rüter et al. (2023). After 4 minutes of sonication in an ultrasonic bath (Bandelin Sonorex Super 10 P, type DK 102 P, Berlin, Germany) with 35 kHz and 60 W/L, the shoots were incubated in the bacterial solution in the dark for one hour. Shoots were dried with sterile filter paper and then placed onto ½ MS medium with 3% sucrose, 8 g/L Plant Agar, 100 mM acetosyringone and kept in the dark at 24°C for 3 days. Afterwards, the shoots were washed 30 min at 100 rpm in ½ MS liquid medium with 500 mg/L cefotaxime, dried again with sterile filter paper and transferred to ½ MS medium with 3% sucrose, 8 g/L Plant Agar and 500 mg/L cefotaxime. The shoots were further cultivated at 24℃ with a 16 h photoperiod. After 4 weeks, hairy roots formed on the shoots were cut and placed onto shoot regeneration medium consisting of full MS medium with 8 g/L Plant Agar, 3% sucrose, 8.9 µM BAP, 1.5 µM IBA, 1.4 µM GA3 and 2.3 µM TDZ. Ri shoots formed after a few months.

**ND Ri genotypes:**

Hairy roots were induced on ND leaf explants following the protocol from Rüter et al. (2023). After formation, hairy roots were placed onto root propagation medium composed of ½ MS salts with vitamins, 3% sucrose, 3.5 g/L gelrite (Duchefa, Haarlem, The Netherlands), and 100 mg/L timentin at pH 5.8. Hairy root cultures were maintained in dark conditions at 22 °C ± 2 °C, sub-cultured every 2 months and Ri shoots emerged after 1 – 2 years.

Table A. Overview of all experiments/batches (numbered chronologically) and the morphological, gene expression, and hormonal analyses performed on different plant organs.

| **Organs** | **Morphology** | **Gene expression** | **Hormones** |
| --- | --- | --- | --- |
| In vitro roots | 1, 2 | 3 | 4 |
| Shoots, leaf parameters, tubers | 5 | - | - |
| Dry masses, root hair statuses | 6 | - | - |
| Leaves, stems | - | 7 | 7 |

Dry masses and root hair status

Plant propagation and rooting procedures followed the previously described methods with the modification that rooting was performed using 9 g/L Phyto Agar (Duchefa, Haarlem, The Netherlands). Plants were then transferred to peat substrate (type VMV800: 70% peat H2-H5, clay and perlite, 100 mg/L N + 140 mg/L P_2_O_5_ + 170 mg/L K_2_O + 80 mg/L Mg, Einheitserde Werkverband e.V., Sinntal-Altengronau, Germany) and placed within a foil tent inside a greenhouse for acclimatization. Acclimatization conditions included a 16-hour photoperiod with temperatures maintained at 21°C. Humidity levels were gradually reduced over four weeks. Twelve plants per genotype were transferred to 13 cm diameter pots with MyPex foil covering the drainage holes. Pots were filled with a sandy soil from Heidgraben (Germany) that had been sieved at 8 mm and gamma-irradiated with a minimum dosage of 10 kGy (Synergy Health Radeberg GmbH, Radeberg, Germany) to exclude pathogens. Two g/L of slow-release fertilizer (Osmocote Exact: 16N-9P-12K, Everris International B.V., Geldermalsen, The Netherlands) was incorporated into the soil. Greenhouse conditions were maintained under a 12-hour day/12-hour night cycle at 21°C during the day and 18°C at night. After eight weeks of growth, samples for root hair analysis were collected and shoots and roots were separated. Plant materials were dried at 70°C for four days and subsequently weighed. These data are recorded in file S1 Raw_Data within the sheet "In_vivo_dryMass". For root hair analysis, six plants per genotype with an average height were selected. Ten 2 cm root tips of the first order (smallest lateral roots) per plant were carefully freed from soil residues using water and forceps and preserved in 70% isopropanol at 4°C. Root hair parameters were analyzed by first rehydrating root tips in water and placing them on microscope slides. Root tips were immersed in 0.05% toluidine blue solution for 5 min and subsequently washed twice with water before microscopic examination at 20x magnification with a digital microscope (VHX-7000, Keyence, Osaka, Japan). Analyses of root hair number, root hair area, root body area without root hairs, and root hair length of the first 5 mm of each tip were conducted using Fiji with a custom-developed Fiji macro, which is available after contact. Root hair raw data are presented in the sheet "In_vivo_rootHairs".

Table B. Overview of actin primers used for gDNA contamination control.

| **Sequence** | **Species** | **cDNA fragment length [bp]** | **Reference** |
| --- | --- | --- | --- |
| F: CGAGGAAGATCTGGCATCA R: AGGAGCTGCTCTTGGCAGT | Rose | 460 | Hattendorf and Debener (2007) |
| F: CATCCTGTCCTCCTAACTGAAGCC R: TCACCAGAGTCCAACACAATACCG | Potato | 170 | Saubeau et al. (2016) |

Table C. Overview of primers used for gene expression analysis.

| **Gene** | **Sequence** | **Efficiency** | **R2** | **Species** | **cDNA fragment length [bp]** | **Reference** |
| --- | --- | --- | --- | --- | --- | --- |
| *rolA* | F: CCAATCTGAGCACCACTCCT R: AATCCCGTAGGTTTGTTTCG | 70 | 0.993 | Rose, Potato | 153 | Lütken et al. (2012) |
| *rolB* | F: GATATCCCGAGGGCATTTTT R: GAATGCTTCATCGCCATTTT | 84 | 0.993 | Rose, Potato | 182 | Lütken et al. (2012) |
| *rolC* | F: CAATAGAGGGCTCAGGCAAG R: CCTCACCAACTCACCAGGTT | 80 | 0.981 | Rose, Potato | 202 | Lütken et al. (2012) |
| *aux2* | F: TGCGCCTTGAAGTAACTGTG R: AGCTTTCCGACTGCCATCTA | 87 | 0.987 | Rose, Potato | 233 | Lütken et al. (2012) |
| *OXA1* | F: GCGATTCTGAAGCCAAAACT R: TTGTTGACCTTCAGCCACAG | 98 | See reference | Potato | 87 | Castro-Quezada et al. (2013) |
| *RPN7* | F: TTGGGGTGTCTGAGGATTTC R: CATTCTTTGCATCAGGACGA | 100 | See reference | Potato | 123 | Castro-Quezada et al. (2013) |
| *SAND* | F: GTGTTGAGGAGTTGCCTCTTG R: AACCTGTCGGGAGAATCTGTT | 98 | See reference | Rose | 97 | Klie and Debener (2011) |
| *TIP* | F: GAATCCACGGCTGGGAAA R: CAGTTCGTGGGTGGAGGAGTT | 93 | See reference | Rose | 65 | Klie and Debener (2011) |

Plant hormone analysis

**In vitro roots, at the Nicolaus Copernicus University in Toruń, Poland:**

The samples were ground in liquid nitrogen using a CryoMill (Retsch GmbH). For the phytohormone analysis in roots, the homogenates were suspended in 1.7 mL solution composed of 80% (v/v) Acetonitrile (ACN), containing 1 mM 2,6-di-tert-butyl-4-methylphenol, 5% (v/v) formic acid. The samples were spiked with a mixture of deuterated internal standards. The standard mixture consisted of 8 ng d5-JA, 8 ng d4-SA, 4 ng d6-ABA, 8 ng d2-JAMe, 4 ng d2-IAA, 1 ng d2-IP and 1 ng d5-tZ. After the addition of internal standards, the homogenates were agitated (170 rpm) overnight at 4°C. Then, 60 mg of MgSO_4_ and 20 mg of NaCl were added and samples were vortexed and agitated (170 rpm) for 30 min at room temperature, for partition of water and acetonitrile phases. The samples were centrifuged at 20,000 × g for 10 min at 4°C to separate water and ACN phases. The upper, ACN phase was transferred to new tubes. Then, 80 mg of anhydrous Na_2_SO_4_ was added to samples to remove the remaining trace amount of water. Next, the samples were agitated, centrifuged, and transferred to new tubes. After that, the ACN phase was evaporated to dryness under a nitrogen stream at 40°C. The remaining residue was suspended in 1 mL of 1 M formic acid and loaded onto pre-conditioned (i.e. washed with 1 mL of 100% (v/v) methanol and 2 mL of 1 M formic acid) Chromabond SPE column (Macherey-Nagel, Düren, Germany). After loading the samples, the columns were washed with 2 mL of 1 M formic acid and the retained analytes were eluted with 0.5 mL of 80% (v/v) methanol. The eluates were lyophilized, then suspended in 100 µl of 35% (v/v) methanol with 0.1% formic acid (w/v) and analyzed with LC-MS/MS.

Samples were analyzed on a Nexera XR UHPLC (Shimadzu, Kyoto, Japan) coupled to a LCMS-8045 integrated systems (Shimadzu, Kyoto, Japan) equipped with a binary gradient solvent pump, a triplequadrupole mass analyzer with electrospray ionization (LC-ESI-MS/MS). Chromatographic separation was achieved on a Ascentis Express C-18 column (2.7 μm, 100 × 2.1 mm, Supelco, Bellefonte, PA, USA), maintained at 35°C, using 0.1% formic acid in water (mobile phase A) and methanol with 0.1% formic acid (mobile phase B) acid, at a flow rate of 0.35 mL/min. The gradient started at 35% B, then was raised linearly to 90% B during the next 4 min, and then to 100% B during the next 2 min. Positive and negative ion mode detection was performed using multiple reaction monitoring (MRM) at m/z 211.1>133.3 transition for JA, 214.2>134.3 for d5-JA, 137.0>93.0 for SA, 141.0>97.0 for d4-SA, 263.3->153.2 for ABA, 269.3>159.2 for d6-ABA, 225.1>151.2 for JAMe and 227.1>153.1 for d2-JAMe, 176.2>130.3 for IAA, 178.2>132.3 for d2-IAA, 220.1>202.1 for tZ, 225.3>137.2 for d5-tZ, 204.2>148.1 for iP and 210.2>137.2 for d2-iP. The peak area of the diagnostic product ion and precursor ion under optimized conditions was used for quantification. Data acquisition was performed with LabSolutions software 5.8 (Shimadzu, Kyoto, Japan).

For roots, the detection limits of the hormones IAA, ABA, SA, JA, iP and JAMe were set to 0.625, 0.25, 17.5, 6.25, 0.125, 0.375 ng/g of sample dry mass, respectively.

**Greenhouse stems and leaves, at the Polish Academy of Sciences, Kraków, Poland:**

All reagents used for this extraction and HPLC analysis were sourced from Sigma-Aldrich Sp. z o.o. (Poznań, Poland). Analytical standards, comprising both unlabeled and stable isotope-labeled phytohormones, were obtained from Olchemim (Olomouc, Czech Republic). Plant material was lyophilized and ground to a fine powder prior to analysis. An internal standard solution was added to each sample and included D_5_-indole-3-acetic acid, D_6_-abscisic acid, D_4_-salicylic acid, and D_6_-jasmonic acid, along with ^15^N_4_-trans-zeatin. Phytohormones were extracted twice using a methanol:water:formic acid mixture (15:4:1, v/v/v), following the protocol of Dobrev and Kamínek (2002), with modifications based on the procedure described by Štefančič et al. (2007). Combined extracts were evaporated to dryness and reconstituted in 1 M formic acid. The phytohormone extracts were subjected to solid-phase extraction (SPE) using Oasis MCX cartridges (30 mg, Waters). Acidic compounds, including auxins and stress-related phytohormones, were eluted with methanol, while basic phytohormones (cytokinins) were recovered using 0.35 M ammonia in 60% methanol. Both eluates were dried under vacuum and redissolved in 100 µL of methanol. Chromatographic separation was performed on a Supelco Ascentis RP-Amide column (75 mm × 4.6 mm, 2.7 µm particle size). For the separation of acidic phytohormones, the mobile phase consisted of 0.1% formic acid in water (solvent A) and a 1:1 (v/v) acetonitrile:methanol mixture (solvent B). In the case of basic phytohormones, solvent A was replaced with 0.001% acetic acid in water, while solvent B remained the same. Gradient elution was applied at a flow rate of 0.5 mL/min. Detection was carried out using an Agilent 1260 HPLC system coupled to an Agilent 6410 Triple Quadrupole mass spectrometer equipped with an electrospray ionization (ESI) source. The instrument operated in multiple reaction monitoring (MRM) mode, tracking the two most abundant product ions for each analyte. Quantification was based on calibration curves constructed using external standards corresponding to each target compound.

In leaf and stem analyses, we detected cZR, tZR, DHZR, iP, IAA, ABA, SA, and JA, as these tissues contained sufficiently high hormone concentrations. The remaining compounds were not detected, and their limit of detection was estimated, based on standard analyses, at approximately 10 ng/g of sample dry mass.

REFERENCES

Castro-Quezada, P., Aarrouf, J., Claverie, M., Favery, B., Mugniéry, D., Lefebvre, V., et al. (2013). Identification of Reference Genes for Normalizing RNA Expression in Potato Roots Infected with Cyst Nematodes. *Plant Mol. Biol. Rep.* 31, 936–945. doi: 10.1007/s11105-013-0566-3

Dobrev, P. I., and Kamínek, M. (2002). Fast and efficient separation of cytokinins from auxin and abscisic acid and their purification using mixed-mode solid-phase extraction. *J. Chromatogr. A* 950, 21–29. doi: 10.1016/S0021-9673(02)00024-9

Klie, M., and Debener, T. (2011). Identification of superior reference genes for data normalisation of expression studies via quantitative PCR in hybrid roses (*Rosa* hybrida). *BMC Res. Notes.* 4, 518. doi: 10.1186/1756-0500-4-518

Lütken, H., Jensen, E. B., Wallström, S. V., Müller, R., and Christensen, B. (2012). DEVELOPMENT AND EVALUATION OF A NON-GMO BREEDING TECHNIQUE EXEMPLIFIED BY *KALANCHOË*. *Acta. Hortic.*, 51–58. doi: 10.17660/ActaHortic.2012.961.3

Rüter, P., Wehrenberg, F., Bartels, J., Debener, T., and Winkelmann, T. (2023). Optimization of *Rhizobium rhizogenes* -mediated transformation for a diversity set of rose genotypes. *Acta. Hortic.*, 225–234. doi: 10.17660/ActaHortic.2023.1383.27

Štefančič, M., Štampar, F., Veberič, R., and Osterc, G. (2007). The levels of IAA, IAAsp and some phenolics in cherry rootstock ‘GiSelA 5’ leafy cuttings pretreated with IAA and IBA. *Sci. Hortic.* 112, 399–405. doi: 10.1016/j.scienta.2007.01.004
