# Supplementary material for: Morphological and hormonal diversity in rose (Rosa hybrida L.) and potato (Solanum tuberosum L.) Ri genotypes: A comparative study
Source: PLoS One. 2026 Apr 15;21(4):e0345941. doi: 10.1371/journal.pone.0345941 (PMC13082669; doi:10.1371/journal.pone.0345941)
Supplement: S4 File — Supplementary results. Graphical presentations of morphological, gene expression and hormone data, gene expression ANOVA table, correlation tables of significant correlation across all Ri genotypes, correlations with at least one significant correlation in one cultivar, correlations with the same trend across all three cultivars. (DOCX) [file pone.0345941.s004.docx]

Supplementary Results


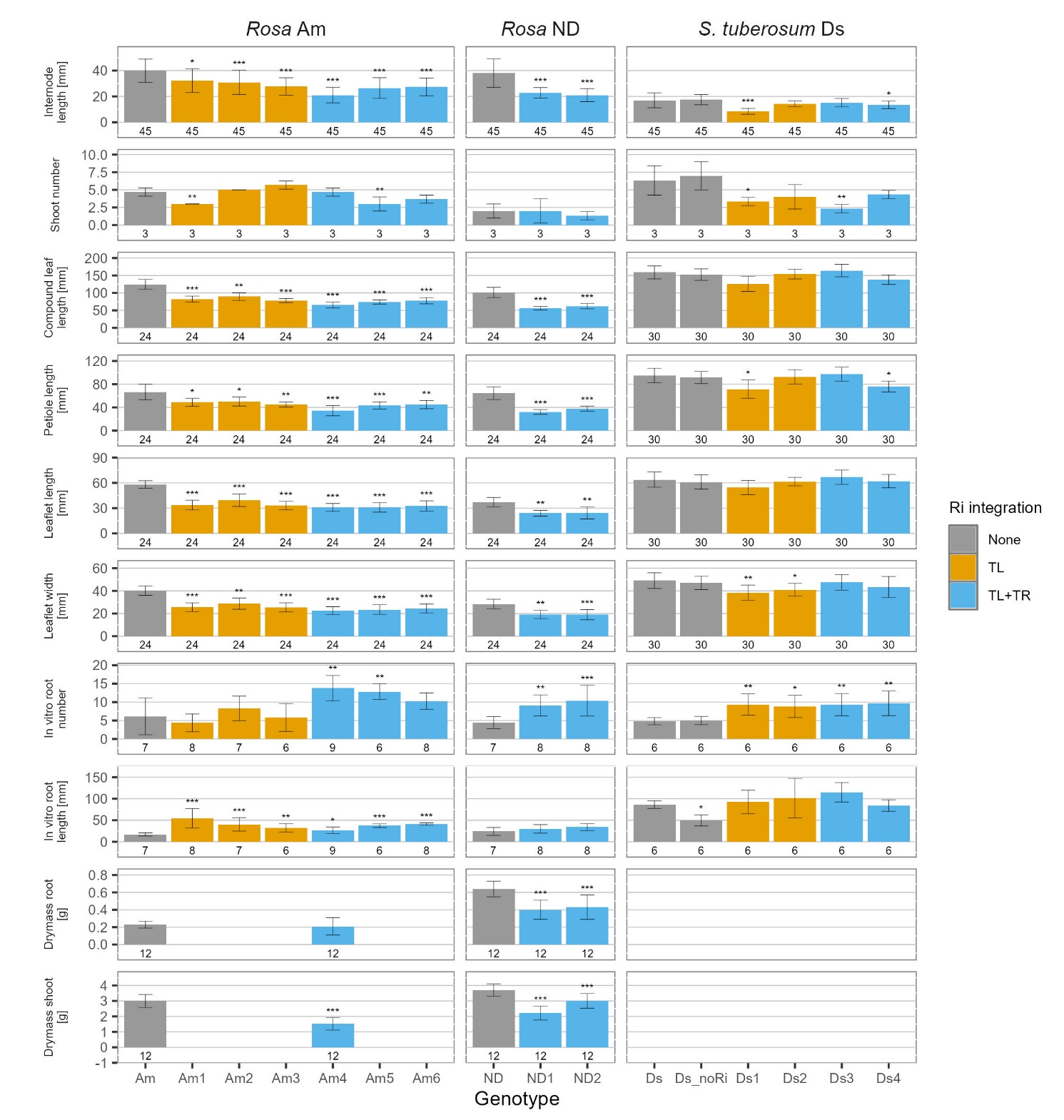


Fig A. Morphological parameters. Shown are means ± standard deviations, n is shown below each bar. Asterisks indicate a significant difference to the wildtype according to Dunnett’s test with p < 0.05 = *, p < 0.01 = ** and p < 0.001 = ***. Fitting of regression models is described in the Materials and Methods section.


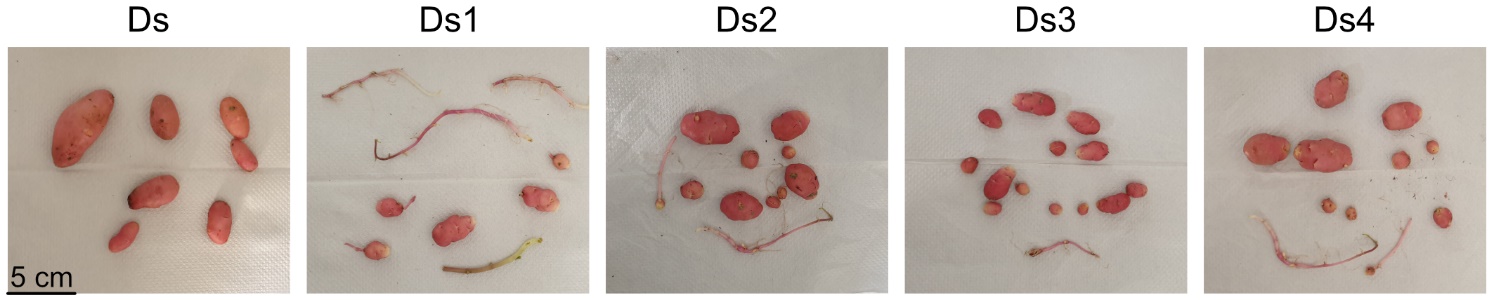


Fig B. Tubers of *S. tuberosum* from greenhouse grown wildtype and Ri genotype plants. Shown are all tubers and stolons of one representative plant per genotype.


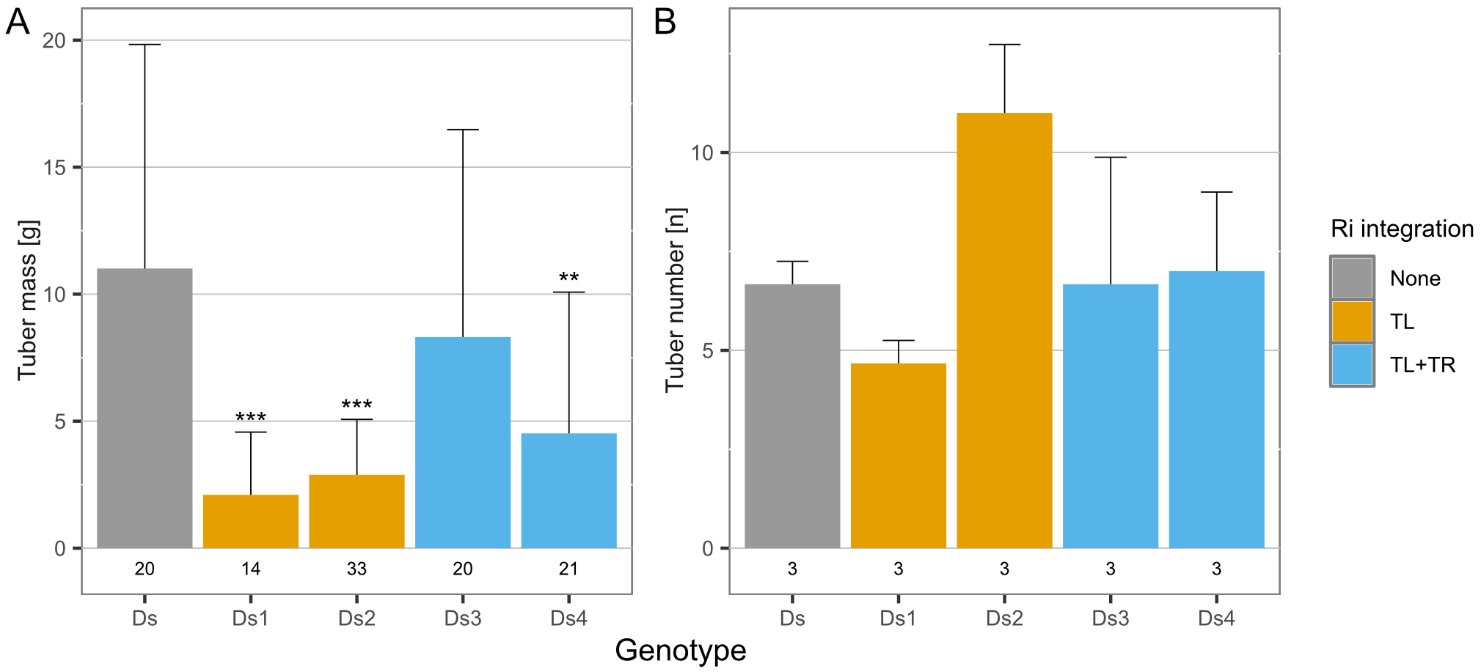


Fig C. *S. tuberosum* tuber parameters from greenhouse grown wildtype and Ri genotype plants. Shown are means + standard deviations, n is shown below each bar. Asterisks indicate a significant difference to the wildtype according to Dunnett’s test with *p* < 0.05 = *, *p* < 0.01 = ** and *p* < 0.001 = ***. (A) Tuber mass, n = Tuber number. For statistical evaluation, a linear model with genotype as fixed effect was fitted to Box-Cox-transformed data. (B) Tuber number, n = Number of plants. For statistical evaluation, a generalized linear model with a Quasi-Poisson distribution was fitted with genotype as fixed effect.


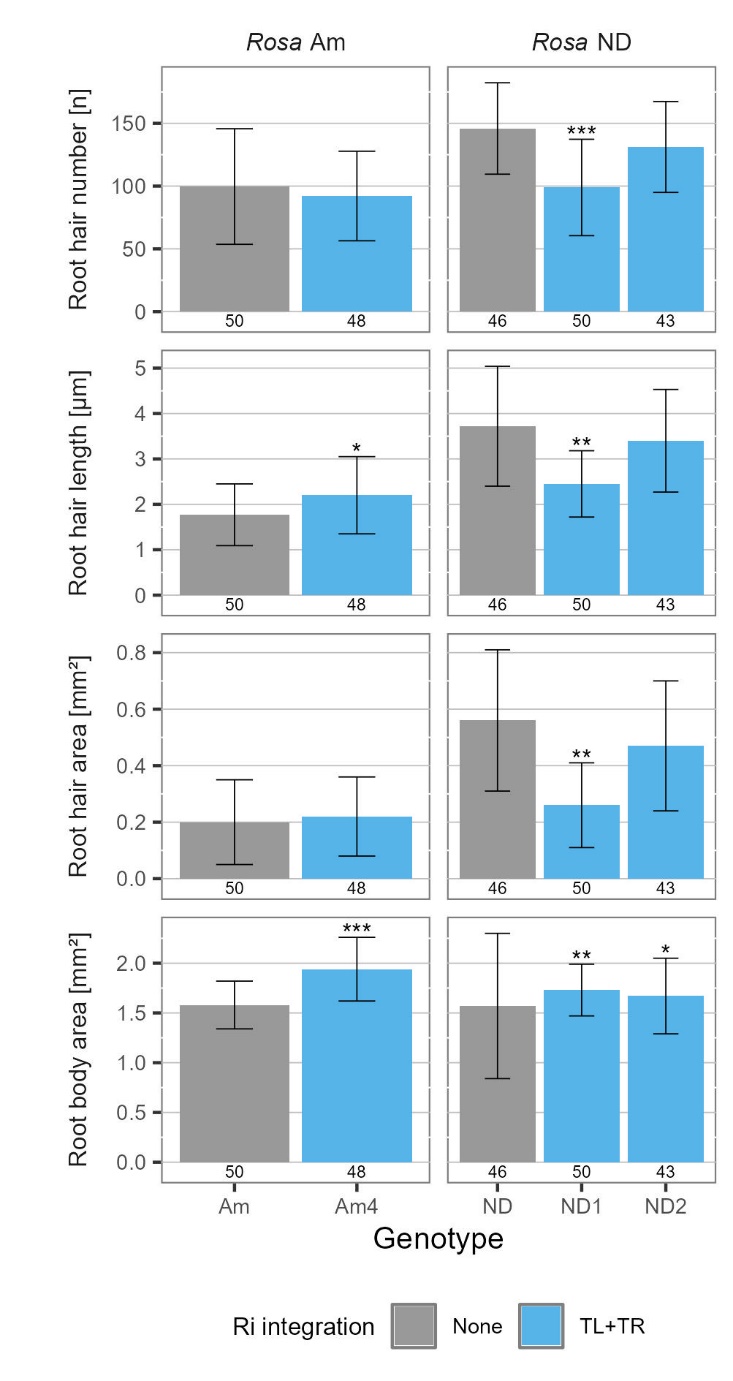


Fig D. Root hair parameters of rose Ri genotypes (blue) and their respective wildtypes (grey). Shown are two cultivars, Am and ‘New Dawn’ (ND), with means ± standard deviations. The number of root tips is shown below each bar. Five mm of approximately 8 root tips of 6 plants per genotype were analyzed. Asterisks indicate significant differences compared to the wildtype according to Dunnett’s test (*p* < 0.05 = *, *p* < 0.01 = **, *p* < 0.001 = ***) using regression models, as explained in the Materials and Methods section.

Table A. ANOVA results of expression analysis. Shown are *p* values for indications of significantly different expression levels between Ri genotypes for each gene, cultivar and organ. Significance thresholds are *p* < 0.05 = *, *p* < 0.01 = ** and *p* < 0.001 = ***. NA = not analyzed.

|  | ***Rosa* Am** | | | ***Rosa* ND** | | | ***S. tuberosum* Ds** | | |
| --- | --- | --- | --- | --- | --- | --- | --- | --- | --- |
|  | **Leaf** | **Stem** | **Root** | **Leaf** | **Stem** | **Root** | **Leaf** | **Stem** | **Root** |
| ***rolA*** | 0.1698 | 0.0037  ** | 0.0014  ** | 0.7277 | 0.2076 | 0.1236 | 0.1561 | 0.0001  *** | 0.0890 |
| ***rolB*** | 0.0014  ** | 0.0729 | 0.0021  ** | 0.4323 | 0.4118 | 0.0617 | 0.4045 | 0.0024  ** | 0.0481  * |
| ***rolC*** | 0.8508 | 0.2797 | 0.0521 | 0.1247 | 0.9491 | 0.1234 | 0.0112  * | 0.0098  ** | 0.1071 |
| ***aux2*** | 0.0118  * | 0.7838 | NA | 0.0090  ** | 0.6841 | NA | NA | NA | NA |


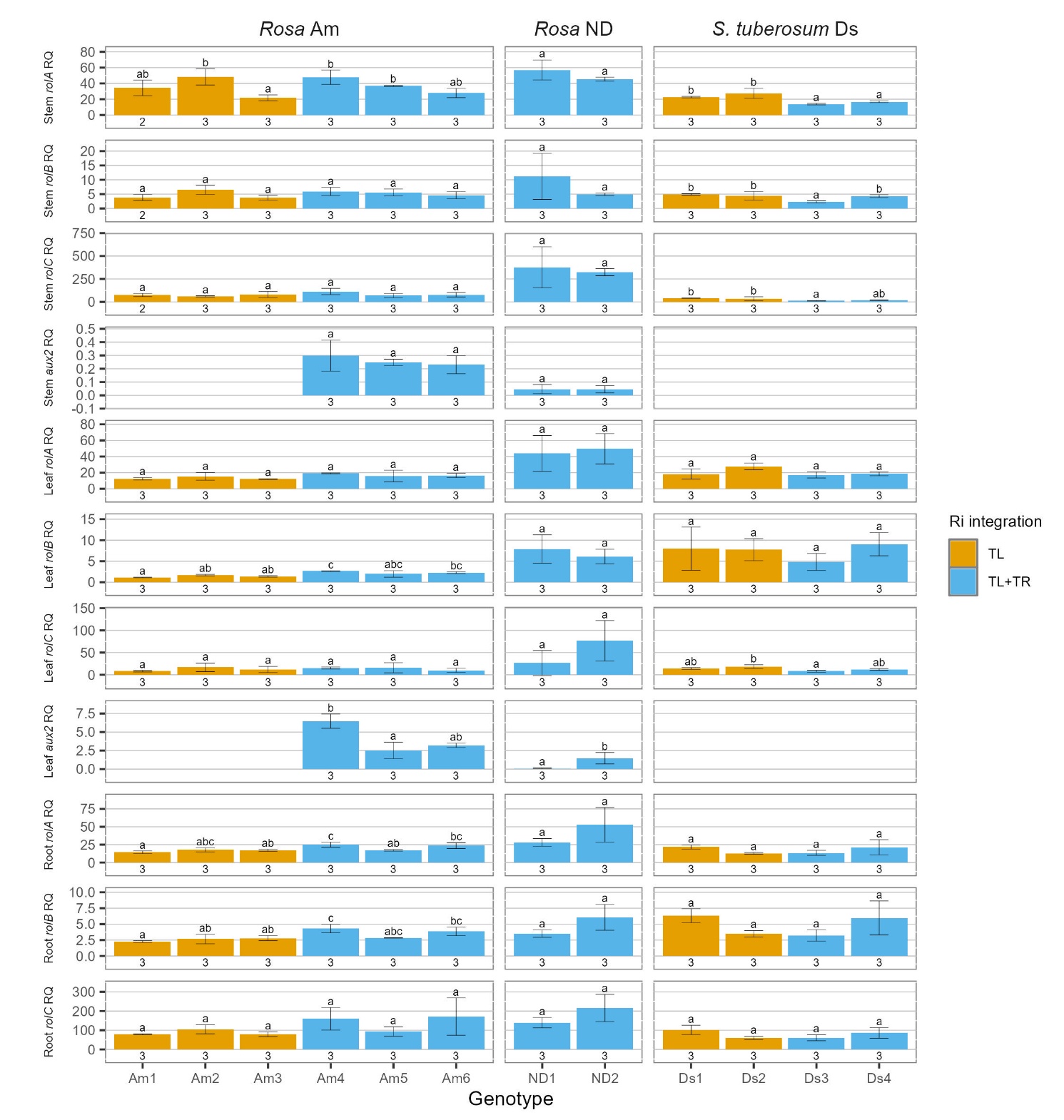


Fig E. Relative quantity (RQ) of expression levels to internal reference genes for all organs, and the analyzed T-DNA genes. Shown are means ± standard deviations, n (plant) is shown below each bar. Significance letters indicate significant differences according to Tukey’s test between genotypes for each cultivar, organ and gene. Fitting of regression models is described in the Materials and Methods section.


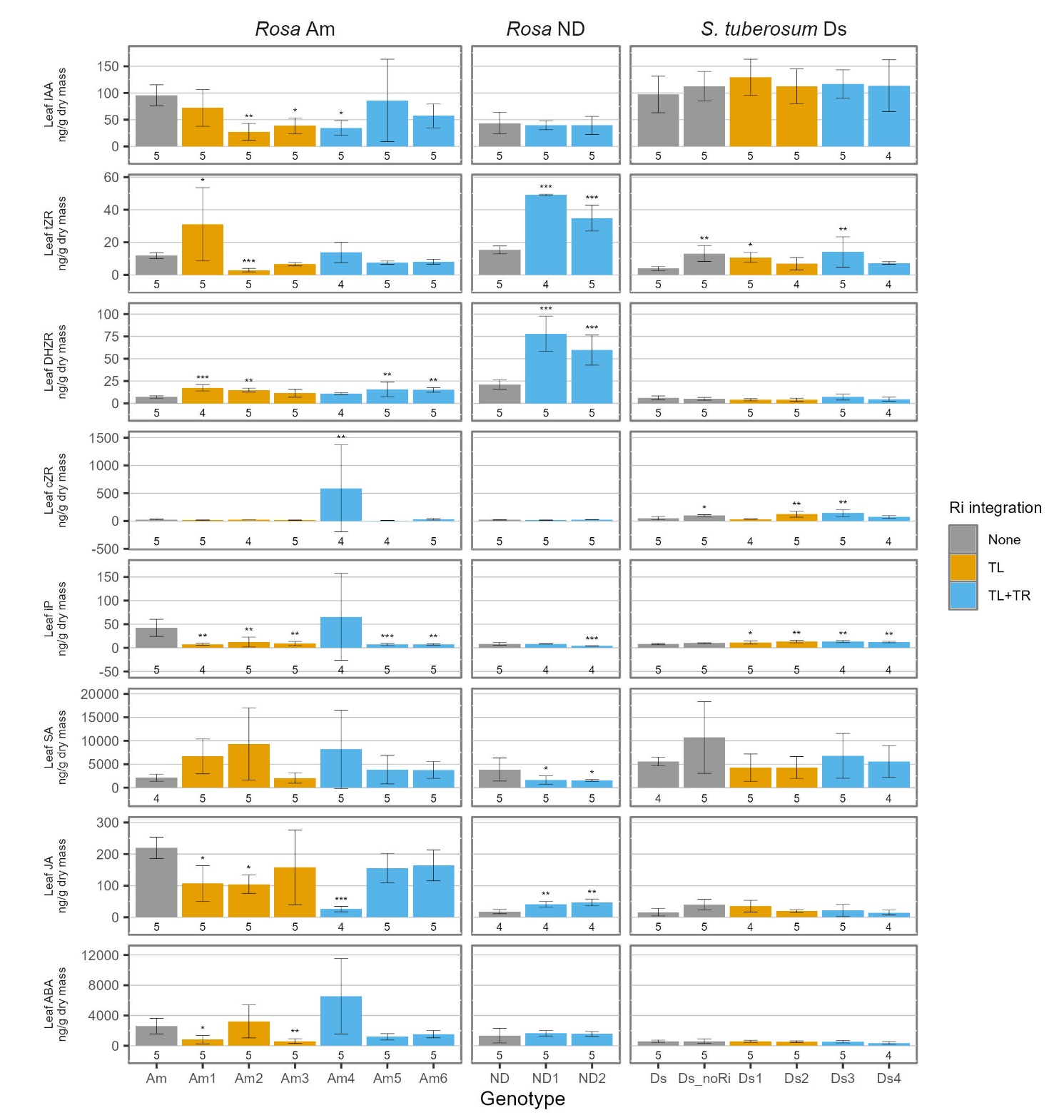


**Fig F. Hormone concentrations of leaf samples.** Shown are means ± standard deviations, n is shown below each bar. Asterisks indicate a significant difference to the wildtype according to Dunnett’s test with p < 0.05 = *, p < 0.01 = ** and p < 0.001 = ***. Fitting of regression models is described in the Material and Method section.

**
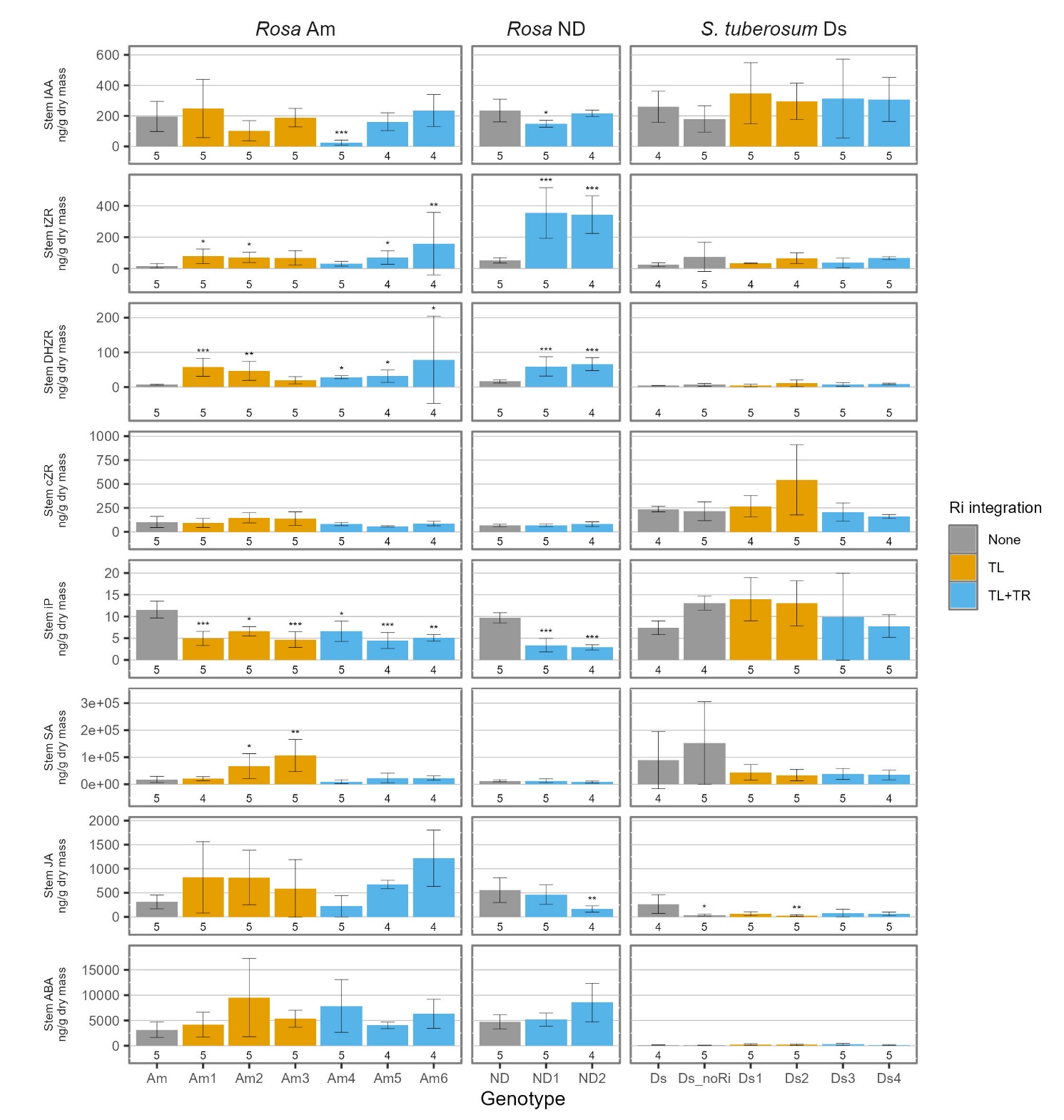
**

**Fig G. Hormone concentrations of stem samples.** Shown are means ± standard deviations, n is shown below each bar. Asterisks indicate a significant difference to the wildtype according to Dunnett’s test with p < 0.05 = *, p < 0.01 = ** and p < 0.001 = ***. Fitting of regression models is described in the Materials and Methods section.


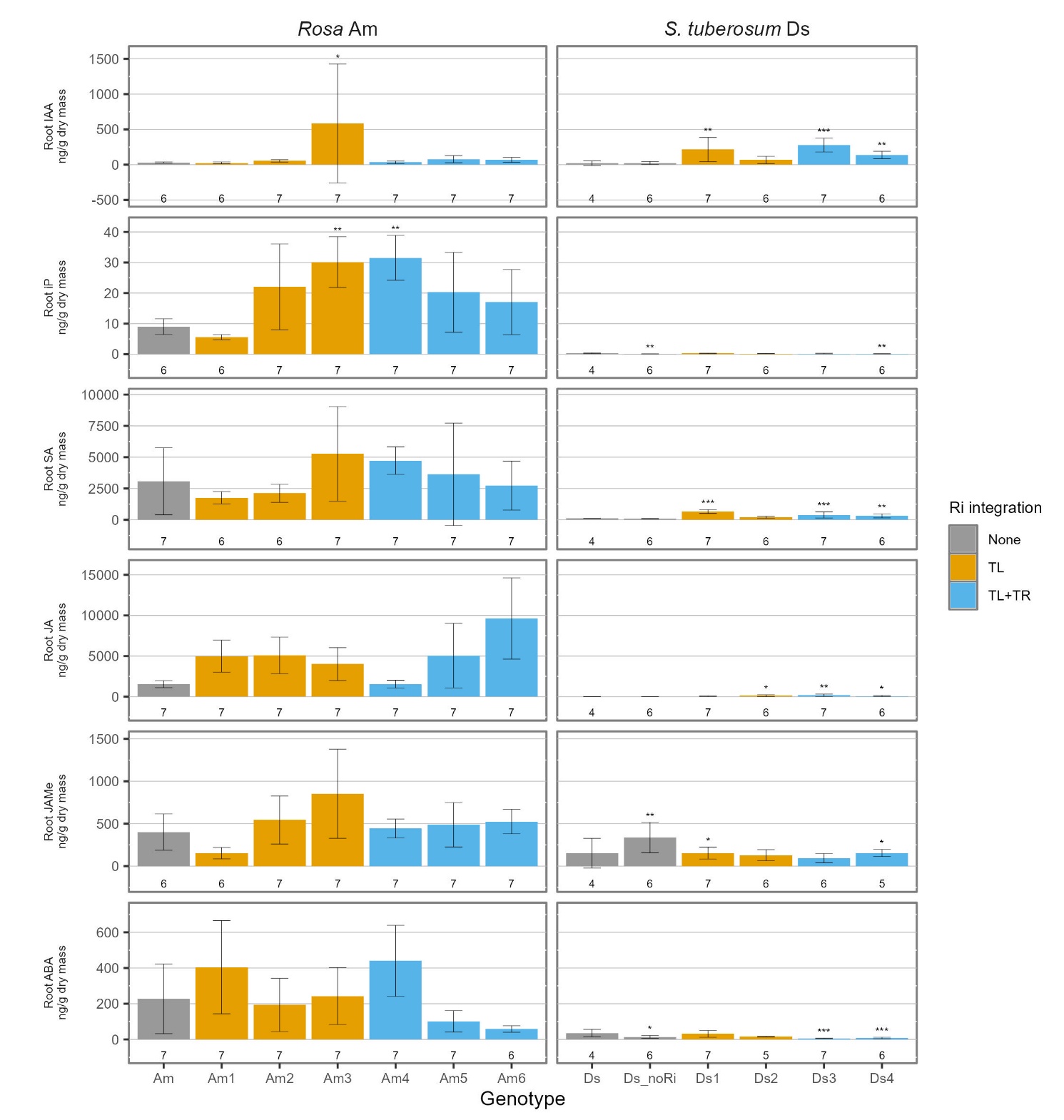


**Fig H. Hormone concentrations of root samples.** Shown are means ± standard deviations, n (plant) is shown below each bar. Asterisks indicate a significant difference to the wildtype according to Dunnett’s test with p < 0.05 = *, p < 0.01 = ** and p < 0.001 = ***. Fitting of regression models is described in the Material and Method section.

Table B. Significant correlations across all rose and potato Ri genotypes of T-DNA gene expressions, morphological and hormonal trait ratios relative to the respective wildtype. Separate correlation analyses were performed with the Spearman algorithm and a significance threshold of 0.05 for the organs root (brown), stem (yellow) and leaf (green). Positive correlations are shown in blue, negative in red. For morphological data, simple ratios of means per genotype were used (1 for wildtypes), for hormonal data the log2-fold-change of means (0 for wildtypes). For expression data, the mean RQ value was scaled between 0.1 and 1 (0 for wildtypes). N = number of genotypes.

| **Comparisons** | **Parameter 1** | **Parameter 2** | **Organ** | **r** | ***p*** | **n** |
| --- | --- | --- | --- | --- | --- | --- |
| Expression –  Expression | *rolA* | *rolB* | Leaf | 0.79 | 0.001 | 13 |
|  | *rolA* | *rolB* | Root | 0.9 | 2.15e-05 | 13 |
|  | *rolA* | *rolB* | Stem | 0.79 | 0.001 | 13 |
|  | *rolA* | *rolC* | Leaf | 0.77 | 0.002 | 13 |
|  | *rolA* | *rolC* | Root | 0.92 | 6.41e-06 | 13 |
|  | *rolA* | *rolC* | Stem | 0.57 | 0.044 | 13 |
|  | *rolB* | *rolC* | Leaf | 0.83 | 4.05e-04 | 13 |
|  | *rolB* | *rolC* | Root | 0.91 | 1.99e-05 | 13 |
|  | *rolB* | *rolC* | Stem | 0.72 | 0.005 | 13 |
| Hormones –  Morphology | IAA | Compound leaf length [mm] | Leaf | 0.61 | 0.028 | 13 |
|  | IAA | Leaflet length [mm] | Leaf | 0.72 | 0.005 | 13 |
|  | IAA | Leaflet width [mm] | Leaf | 0.72 | 0.006 | 13 |
|  | IAA | Shoot number | Stem | -0.66 | 0.014 | 13 |
|  | DHZR | Compound leaf length [mm] | Leaf | -0.7 | 0.01 | 13 |
|  | DHZR | Leaflet length [mm] | Leaf | -0.57 | 0.043 | 13 |
|  | DHZR | Petiole length [mm] | Leaf | -0.73 | 0.005 | 13 |
|  | iP | Leaflet length [mm] | Leaf | 0.65 | 0.016 | 13 |
|  | iP | Leaflet width [mm] | Leaf | 0.6 | 0.034 | 13 |
|  | SA | In vitro root length [mm] | Root | -0.73 | 0.015 | 11 |
|  | SA | Shoot number | Stem | 0.61 | 0.027 | 13 |
| Hormones –  Hormones | IAA | JA | Leaf | 0.74 | 0.004 | 13 |
|  | IAA | SA | Leaf | -0.59 | 0.035 | 13 |
|  | IAA | SA | Root | 0.8 | 0.005 | 11 |
|  | IAA | iP | Leaf | 0.57 | 0.042 | 13 |
|  | IAA | tZR | Leaf | 0.65 | 0.017 | 13 |
|  | tZR | DHZR | Stem | 0.83 | 7.78e-04 | 13 |
|  | tZR | iP | Stem | -0.75 | 0.003 | 13 |
|  | cZR | iP | Leaf | 0.75 | 0.004 | 13 |
|  | DHZR | iP | Leaf | -0.67 | 0.015 | 13 |
|  | DHZR | iP | Stem | -0.61 | 0.026 | 13 |
|  | iP | JAMe | Root | 0.81 | 0.003 | 11 |
|  | SA | DHZR | Stem | 0.59 | 0.036 | 13 |
|  | SA | JA | Leaf | -0.9 | 2.83e-05 | 13 |
|  | SA | JA | Stem | 0.88 | 8.06e-05 | 13 |
|  | SA | iP | Stem | -0.65 | 0.017 | 13 |
|  | JA | DHZR | Stem | 0.69 | 0.009 | 13 |
|  | JA | tZR | Leaf | 0.6 | 0.032 | 13 |
|  | ABA | JA | Root | -0.89 | 2.58e-04 | 11 |
| Morphology –  Morphology | Compound leaf length [mm] | Leaflet length [mm] | Leaf | 0.89 | 5.58e-05 | 13 |
|  | Compound leaf length [mm] | Leaflet width [mm] | Leaf | 0.87 | 6.82e-05 | 13 |
|  | Compound leaf length [mm] | Petiole length [mm] | Leaf | 0.99 | 6.02e-10 | 13 |
|  | Petiole length [mm] | Leaflet length [mm] | Leaf | 0.83 | 4.79e-04 | 13 |
|  | Petiole length [mm] | Leaflet width [mm] | Leaf | 0.82 | 6.80e-04 | 13 |
|  | Leaflet length [mm] | Leaflet width [mm] | Leaf | 0.99 | 3.36e-10 | 13 |

Table C. Correlations of each cultivar of T-DNA gene expressions, morphological and hormonal trait ratios relative to the respective wildtype with at least one significant correlation. Separate correlation analyses were performed with the Spearman algorithm and a significance threshold of 0.05 for the organs root (brown), stem (yellow) and leaf (green). Positive correlations are shown in blue, negative in red. Significant correlations are marked in bold. For morphological data, simple ratios of means per genotype were used (1 for wildtypes), for hormonal data the log2-fold-change of means (0 for wildtypes). For expression data, the mean RQ value was scaled between 0.1 and 1 (0 for wildtypes). N = number of genotypes.

|  |  |  |  | **Am** |  |  | **ND** |  |  | **Ds** |  |  |
| --- | --- | --- | --- | --- | --- | --- | --- | --- | --- | --- | --- | --- |
|  | **Parameter 1** | **Parameter 2** | **Organ** | **r** | ***p*** | **n** | **r** | ***p*** | **n** | **r** | ***p*** | **n** |
| Expression - Morphology | *rolA* | Compound leaf length [mm] | Leaf | **-0.82** | **0.034** | **7** | -0.5 | 1 | 3 | -0.5 | 0.45 | 5 |
|  | *rolA* | Leaflet length [mm] | Leaf | **-0.79** | **0.033** | **7** | -0.5 | 1 | 3 | -0.6 | 0.35 | 5 |
|  | *rolA* | Leaflet width [mm] | Leaf | **-0.82** | **0.034** | **7** | -1 | 0.333 | 3 | -0.7 | 0.233 | 5 |
|  | *rolA* | Petiole length [mm] | Leaf | **-0.82** | **0.034** | **7** | -0.5 | 1 | 3 | -0.5 | 0.45 | 5 |
|  | *rolB* | Compound leaf length [mm] | Leaf | **-0.86** | **0.024** | **7** | -1 | 0.333 | 3 | -0.8 | 0.133 | 5 |
|  | *rolB* | In vitro root number | Root | **0.79** | **0.048** | **7** | 1 | 0.333 | 3 | 0.67 | 0.219 | 5 |
|  | *rolB* | Leaflet length [mm] | Leaf | **-0.83** | **0.021** | **7** | -1 | 0.333 | 3 | -0.6 | 0.35 | 5 |
|  | *rolB* | Leaflet width [mm] | Leaf | **-0.86** | **0.024** | **7** | -0.5 | 1 | 3 | -0.7 | 0.233 | 5 |
|  | *rolB* | Petiole length [mm] | Leaf | **-0.86** | **0.024** | **7** | -1 | 0.333 | 3 | -0.8 | 0.133 | 5 |
| Expression - Expression | *rolA* | *rolB* | Leaf | **0.96** | **0.003** | **7** | 0.5 | 1 | 3 | 0.7 | 0.233 | 5 |
|  | *rolA* | *rolB* | Root | **0.86** | **0.024** | **7** | 1 | 0.333 | 3 | 0.9 | 0.083 | 5 |
|  | *rolA* | *rolB* | Stem | **0.96** | **0.003** | **7** | 1 | 0.333 | 3 | 0.9 | 0.083 | 5 |
|  | *rolA* | *rolC* | Root | **0.86** | **0.024** | **7** | 1 | 0.333 | 3 | **1** | **0.017** | **5** |
|  | *rolB* | *rolC* | Root | **0.79** | **0.048** | **7** | 1 | 0.333 | 3 | 0.9 | 0.083 | 5 |
|  | *rolB* | *rolC* | Stem | 0.14 | 0.783 | 7 | 1 | 0.333 | 3 | **1** | **0.017** | **5** |
| Hormones - Morphology | SA | Leaflet length [mm] | Leaf | -0.11 | 0.818 | 7 | 0.5 | 1 | 3 | **1** | **0.017** | **5** |
| Hormones - Hormones | IAA | JAMe | Root | **0.86** | **0.024** | **7** | NA | NA | NA | -0.1 | 0.95 | 5 |
|  | tZR | DHZR | Stem | **0.96** | **0.003** | **7** | 0.5 | 1 | 3 | 0.9 | 0.083 | 5 |
|  | DHZR | iP | Leaf | **-0.82** | **0.034** | **7** | -0.5 | 1 | 3 | 0.1 | 0.95 | 5 |
|  | SA | DHZR | Stem | 0.11 | 0.84 | 7 | -0.5 | 1 | 3 | **-1** | **0.017** | **5** |
|  | SA | JA | Leaf | **-0.86** | **0.024** | **7** | -1 | 0.333 | 3 | -0.3 | 0.683 | 5 |
|  | JA | DHZR | Stem | **0.89** | **0.012** | **7** | -1 | 0.333 | 3 | -0.9 | 0.083 | 5 |
|  | JA | tZR | Stem | **0.96** | **0.003** | **7** | -0.5 | 1 | 3 | -0.8 | 0.133 | 5 |
|  | ABA | JA | Root | **-0.82** | **0.034** | **7** | NA | NA | NA | -0.9 | 0.083 | 5 |
| Morphology - Morphology | Compound leaf length [mm] | Leaflet length [mm] | Leaf | **0.99** | **1.46e-05** | **7** | 1 | 0.333 | 3 | 0.9 | 0.083 | 5 |
|  | Compound leaf length [mm] | Leaflet width [mm] | Leaf | **1** | **3.97e-04** | **7** | 0.5 | 1 | 3 | 0.8 | 0.133 | 5 |
|  | Compound leaf length [mm] | Petiole length [mm] | Leaf | **1** | **3.97e-04** | **7** | 1 | 0.333 | 3 | **1** | **0.017** | **5** |
|  | Petiole length [mm] | Leaflet length [mm] | Leaf | **0.99** | **1.46e-05** | **7** | 1 | 0.333 | 3 | 0.9 | 0.083 | 5 |
|  | Petiole length [mm] | Leaflet width [mm] | Leaf | **1** | **3.97e-04** | **7** | 0.5 | 1 | 3 | 0.8 | 0.133 | 5 |
|  | Leaflet length [mm] | Leaflet width [mm] | Leaf | **0.99** | **1.46e-05** | **7** | 0.5 | 1 | 3 | 0.9 | 0.083 | 5 |

Table D. Correlations with the same positive or negative trend of all three cultivars of T-DNA gene expressions, morphological and hormonal trait ratios relative to the respective wildtype. Separate correlation analyses were performed with the Spearman algorithm for the organs root (brown), stem (yellow) and leaf (green). Positive correlations are shown in blue, negative in red. Significant correlations are marked in bold. For morphological data, simple ratios of means per genotype were used (1 for wildtypes), for hormonal data the log2-fold-change of means (0 for wildtypes). For expression data, the mean RQ value was scaled between 0.1 and 1 (0 for wildtypes). N = number of genotypes.

|  |  |  |  | **Am** | | | **ND** | | | **Ds** | | |
| --- | --- | --- | --- | --- | --- | --- | --- | --- | --- | --- | --- | --- |
|  | **Parameter 1** | **Parameter 2** | **Organ** | **r** | ***p*** | **n** | **r** | ***p*** | **n** | **r** | ***p*** | **n** |
| Expression - Morphology | *rolA* | Compound leaf length [mm] | Leaf | **-0.82** | **0.034** | **7** | -0.5 | 1 | 3 | -0.5 | 0.45 | 5 |
|  | *rolA* | In vitro root number | Root | 0.64 | 0.139 | 7 | 1 | 0.333 | 3 | 0.82 | 0.089 | 5 |
|  | *rolA* | Internode length [mm] | Stem | -0.46 | 0.302 | 7 | -0.5 | 1 | 3 | -0.7 | 0.233 | 5 |
|  | *rolA* | Leaflet length [mm] | Leaf | **-0.79** | **0.033** | **7** | -0.5 | 1 | 3 | -0.6 | 0.35 | 5 |
|  | *rolA* | Leaflet width [mm] | Leaf | **-0.82** | **0.034** | **7** | -1 | 0.333 | 3 | -0.7 | 0.233 | 5 |
|  | *rolA* | Petiole length [mm] | Leaf | **-0.82** | **0.034** | **7** | -0.5 | 1 | 3 | -0.5 | 0.45 | 5 |
|  | *rolB* | Compound leaf length [mm] | Leaf | **-0.86** | **0.024** | **7** | -1 | 0.333 | 3 | -0.8 | 0.133 | 5 |
|  | *rolB* | In vitro root number | Root | **0.79** | **0.048** | **7** | 1 | 0.333 | 3 | 0.67 | 0.219 | 5 |
|  | *rolB* | Internode length [mm] | Stem | -0.57 | 0.2 | 7 | -0.5 | 1 | 3 | -0.9 | 0.083 | 5 |
|  | *rolB* | Leaflet length [mm] | Leaf | **-0.83** | **0.021** | **7** | -1 | 0.333 | 3 | -0.6 | 0.35 | 5 |
|  | *rolB* | Leaflet width [mm] | Leaf | **-0.86** | **0.024** | **7** | -0.5 | 1 | 3 | -0.7 | 0.233 | 5 |
|  | *rolB* | Petiole length [mm] | Leaf | **-0.86** | **0.024** | **7** | -1 | 0.333 | 3 | -0.8 | 0.133 | 5 |
|  | *rolC* | Compound leaf length [mm] | Leaf | -0.39 | 0.396 | 7 | -0.5 | 1 | 3 | -0.6 | 0.35 | 5 |
|  | *rolC* | In vitro root number | Root | 0.68 | 0.11 | 7 | 1 | 0.333 | 3 | 0.82 | 0.089 | 5 |
|  | *rolC* | Internode length [mm] | Stem | -0.68 | 0.11 | 7 | -0.5 | 1 | 3 | -0.9 | 0.083 | 5 |
|  | *rolC* | Leaflet length [mm] | Leaf | -0.41 | 0.355 | 7 | -0.5 | 1 | 3 | -0.8 | 0.133 | 5 |
|  | *rolC* | Leaflet width [mm] | Leaf | -0.39 | 0.396 | 7 | -1 | 0.333 | 3 | -0.9 | 0.083 | 5 |
|  | *rolC* | Petiole length [mm] | Leaf | -0.39 | 0.396 | 7 | -0.5 | 1 | 3 | -0.6 | 0.35 | 5 |
| Expression - Hormones | *rolA* | ABA | Stem | 0.68 | 0.11 | 7 | 0.5 | 1 | 3 | 0.1 | 0.95 | 5 |
|  | *rolA* | DHZR | Stem | 0.39 | 0.396 | 7 | 0.5 | 1 | 3 | 0.7 | 0.233 | 5 |
|  | *rolA* | cZR | Leaf | 0.43 | 0.354 | 7 | 0.5 | 1 | 3 | 0.2 | 0.783 | 5 |
|  | *rolA* | tZR | Stem | 0.25 | 0.595 | 7 | 1 | 0.333 | 3 | 0.5 | 0.45 | 5 |
|  | *rolB* | ABA | Stem | 0.75 | 0.066 | 7 | 0.5 | 1 | 3 | 0.2 | 0.783 | 5 |
|  | *rolB* | DHZR | Stem | 0.43 | 0.354 | 7 | 0.5 | 1 | 3 | 0.4 | 0.517 | 5 |
|  | *rolB* | tZR | Stem | 0.29 | 0.556 | 7 | 1 | 0.333 | 3 | 0.3 | 0.683 | 5 |
|  | *rolC* | ABA | Stem | 0.43 | 0.354 | 7 | 0.5 | 1 | 3 | 0.2 | 0.783 | 5 |
|  | *rolC* | DHZR | Stem | 0.11 | 0.84 | 7 | 0.5 | 1 | 3 | 0.4 | 0.517 | 5 |
|  | *rolC* | JA | Stem | -0.14 | 0.783 | 7 | -0.5 | 1 | 3 | -0.7 | 0.233 | 5 |
|  | *rolC* | tZR | Stem | 0.07 | 0.906 | 7 | 1 | 0.333 | 3 | 0.3 | 0.683 | 5 |
| Expression - Expression | *rolA* | *rolB* | Leaf | **0.96** | **0.003** | **7** | 0.5 | 1 | 3 | 0.7 | 0.233 | 5 |
|  | *rolA* | *rolB* | Root | **0.86** | **0.024** | **7** | 1 | 0.333 | 3 | 0.9 | 0.083 | 5 |
|  | *rolA* | *rolB* | Stem | **0.96** | **0.003** | **7** | 1 | 0.333 | 3 | 0.9 | 0.083 | 5 |
|  | *rolA* | *rolC* | Leaf | 0.5 | 0.267 | 7 | 1 | 0.333 | 3 | 0.9 | 0.083 | 5 |
|  | *rolA* | *rolC* | Root | **0.86** | **0.024** | **7** | 1 | 0.333 | 3 | **1** | **0.017** | **5** |
|  | *rolA* | *rolC* | Stem | 0.11 | 0.84 | 7 | 1 | 0.333 | 3 | 0.9 | 0.083 | 5 |
|  | *rolB* | *rolC* | Leaf | 0.57 | 0.2 | 7 | 0.5 | 1 | 3 | 0.6 | 0.35 | 5 |
|  | *rolB* | *rolC* | Root | **0.79** | **0.048** | **7** | 1 | 0.333 | 3 | 0.9 | 0.083 | 5 |
|  | *rolB* | *rolC* | Stem | 0.14 | 0.783 | 7 | 1 | 0.333 | 3 | **1** | **0.017** | **5** |
| Hormones - Morphology | tZR | Internode length [mm] | Stem | -0.04 | 0.963 | 7 | -0.5 | 1 | 3 | -0.4 | 0.517 | 5 |
|  | tZR | Leaflet width [mm] | Leaf | -0.14 | 0.783 | 7 | -0.5 | 1 | 3 | -0.3 | 0.683 | 5 |
|  | DHZR | Internode length [mm] | Stem | -0.14 | 0.783 | 7 | -1 | 0.333 | 3 | -0.3 | 0.683 | 5 |
|  | DHZR | Shoot number | Stem | -0.51 | 0.243 | 7 | -0.87 | 0.333 | 3 | -0.2 | 0.783 | 5 |
|  | iP | Compound leaf length [mm] | Leaf | 0.21 | 0.662 | 7 | 0.5 | 1 | 3 | 0.4 | 0.517 | 5 |
|  | iP | Leaflet length [mm] | Leaf | 0.32 | 0.478 | 7 | 0.5 | 1 | 3 | 0.3 | 0.683 | 5 |
|  | iP | Petiole length [mm] | Leaf | 0.21 | 0.662 | 7 | 0.5 | 1 | 3 | 0.4 | 0.517 | 5 |
|  | SA | Internode length [mm] | Stem | 0.04 | 0.963 | 7 | 0.5 | 1 | 3 | 0.3 | 0.683 | 5 |
|  | SA | Shoot number | Stem | 0.38 | 0.398 | 7 | 0.87 | 0.333 | 3 | 0.2 | 0.783 | 5 |
|  | JA | Internode length [mm] | Stem | 0.18 | 0.713 | 7 | 1 | 0.333 | 3 | 0.6 | 0.35 | 5 |
| Hormones - Hormones | IAA | ABA | Leaf | -0.36 | 0.444 | 7 | -0.5 | 1 | 3 | -0.3 | 0.683 | 5 |
|  | IAA | cZR | Leaf | -0.29 | 0.556 | 7 | -0.5 | 1 | 3 | -0.1 | 0.95 | 5 |
|  | tZR | DHZR | Leaf | 0.07 | 0.906 | 7 | 1 | 0.333 | 3 | 0.3 | 0.683 | 5 |
|  | tZR | DHZR | Stem | **0.96** | **0.003** | **7** | 0.5 | 1 | 3 | 0.9 | 0.083 | 5 |
|  | SA | JA | Leaf | **-0.86** | **0.024** | **7** | -1 | 0.333 | 3 | -0.3 | 0.683 | 5 |
|  | SA | JA | Stem | 0.36 | 0.444 | 7 | 0.5 | 1 | 3 | 0.9 | 0.083 | 5 |
|  | SA | iP | Leaf | 0.21 | 0.662 | 7 | 1 | 0.333 | 3 | 0.3 | 0.683 | 5 |
|  | ABA | cZR | Stem | 0.25 | 0.595 | 7 | 1 | 0.333 | 3 | 0.3 | 0.683 | 5 |
| Morphology - Morphology | Internode length [mm] | Shoot number | Stem | 0.11 | 0.816 | 7 | 0.87 | 0.333 | 3 | 0.3 | 0.683 | 5 |
|  | Compound leaf length [mm] | Leaflet length [mm] | Leaf | **0.99** | **1.46e-05** | **7** | 1 | 0.333 | 3 | 0.9 | 0.083 | 5 |
|  | Compound leaf length [mm] | Leaflet width [mm] | Leaf | **1** | **3.97e-04** | **7** | 0.5 | 1 | 3 | 0.8 | 0.133 | 5 |
|  | Compound leaf length [mm] | Petiole length [mm] | Leaf | **1** | **3.97e-04** | **7** | 1 | 0.333 | 3 | **1** | **0.017** | **5** |
|  | Petiole length [mm] | Leaflet length [mm] | Leaf | **0.99** | **1.46e-05** | **7** | 1 | 0.333 | 3 | 0.9 | 0.083 | 5 |
|  | Petiole length [mm] | Leaflet width [mm] | Leaf | **1** | **3.97e-04** | **7** | 0.5 | 1 | 3 | 0.8 | 0.133 | 5 |
|  | Leaflet length [mm] | Leaflet width [mm] | Leaf | **0.99** | **1.46e-05** | **7** | 0.5 | 1 | 3 | 0.9 | 0.083 | 5 |
