# Supplementary material for: Morphological and hormonal diversity in rose (Rosa hybrida L.) and potato (Solanum tuberosum L.) Ri genotypes: A comparative study
Source: PLoS One. 2026 Apr 15;21(4):e0345941. doi: 10.1371/journal.pone.0345941 (PMC13082669; doi:10.1371/journal.pone.0345941)
Supplement: Attachment — Submitted filename: Response to Reviewers.docx [file pone.0345941.s007.docx]

Editor Muhammad Abdul Rehman Rashid

1. Please ensure that your manuscript meets PLOS ONE's style requirements, including those for file naming. The PLOS ONE style templates can be found at

https://journals.plos.org/plosone/s/file?id=wjVg/PLOSOne_formatting_sample_main_body.pdf and

[https://journals.plos.org/plosone/s/file?id=ba62/PLOSOne_formatting_sample_title_authors_affiliations.pdf](https://track.editorialmanager.com/CL0/https:%2F%2Fjournals.plos.org%2Fplosone%2Fs%2Ffile%3Fid=ba62%2FPLOSOne_formatting_sample_title_authors_affiliations.pdf/1/010f019b34bfa979-20dbafa2-3ff6-4dc2-83a8-82a54aed7060-000000/Bp-tTBXB7UF419Muh8uABU0mLIipuS-8TMrsGTZru1A=242)

- First, we would like to thank the editor for accepting this manuscript to be reviewed by the scientific community. We have carefully reviewed the guidelines and ensured that the manuscript now meets PLOS ONE’s style requirements. Specifically, we have corrected the supplementary file names by replacing blanks with underscores ("_") and have adjusted all headings to the correct format.

2. Please note that PLOS One has specific guidelines on code sharing for submissions in which author-generated code underpins the findings in the manuscript. In these cases, we expect all author-generated code to be made available without restrictions upon publication of the work. Please review our guidelines at https://journals.plos.org/plosone/s/materials-and-software-sharing#loc-sharing-code and ensure that your code is shared in a way that follows best practice and facilitates reproducibility and reuse.

- We appreciate the emphasis on reproducibility and open science. We now have added a supplementary zip-file “S5 RStudio Scripts”, in which all author-generated code is made available.

3. We note that the grant information you provided in the ‘Funding Information’ and ‘Financial Disclosure’ sections do not match.

When you resubmit, please ensure that you provide the correct grant numbers for the awards you received for your study in the ‘Funding Information’ section.

- We apologize for this discrepancy. We have updated the Funding Information section to ensure it is accurate, with the information below:

German funding:

- full name of the founder: Deutsche Forschungsgemeinschaft (DFG)
- grant number: WI 2002/6–1
- link: <https://www.dfg.de/>

Polish funding:

- full name of the founder: National Centre for Research and Development (NCBR)
- grant number: SUSCROP/II/RootsPlus/02/2021
- link: <https://www.gov.pl/web/ncbr>

Suscrop funding:

- full name of the founder: European Union’s Horizon 2020 research and innovation program
- grant number: 771134
- link: <https://research-and-innovation.ec.europa.eu>

Reviewer #1: Dear authors

Comments considered must be required for publication. The present article, titled Morphological and hormonal diversity in rose and potato Ri genotypes: A comparative study, is acceptable, but after the requested items.

- We thank the reviewer for their positive assessment of our manuscript and for confirming that it is appropriate for publication. We have addressed the requested corrections as stated below.

This MS appropriate for publication. However, some correction should be performed.

Abstract

-The abstract was inadequate, and further results were furnished.

- We have revised the abstract to include specific quantitative results from our experiments, providing a more detailed summary of our findings.

Introduction

- How were these organisms chosen, and why were other possible species deemed inappropriate for sampling in this work?

- We appreciate this question. As outlined in the introduction, we selected rose and potato because they represent phylogenetically distinct clades (rosids vs. asterids) and possess distinct physiological habits (woody vs. herbaceous). This makes them ideal candidates to evaluate whether the changes induced by wild-strain T-DNA integration are species-dependent or -independent. We have expanded the introduction (line 77-80) to clarify this rationale further.

Material and methods

In vitro root measurements:

- This section should be written briefly.

- We have reviewed this section and shortened it according to your comment. We believe the current level of detail is necessary to ensure the experiments can be accurately reproduced by other researchers.

T-DNA gene expression analysis

- Please elucidate this more scientifically and concisely.

- We have revised this section to be more precise. We have added specific details regarding the digestion of genomic DNA (gDNA) and the controls used to detect potential contamination. Furthermore, we have included additional information regarding the validation of primer specificity via melting curve analysis.

Results

Some sentences need to revision. Please check the entire text. Displayed are ratios which were calculated by dividing the mean of the Ri genotype by the mean of the respective wild-type in page 9 (183-185). The sentence require general revisions in page 17 (lines 344-345).

- We have thoroughly checked the entire text and improved the sentence structure, including the specific lines mentioned, to enhance readability and clarity. In addition, we have commissioned the American Journal Experts to enhance the readability of the manuscript.

Conclusion

This section is poor and needs to improve for your results. Further, there are no comparison between two species.

- Thank you for your comment, which we carefully thought of. Please find an explanation of our motivation and line of arguments for the conclusions section in the following paragraph. We hope that you can agree.
- All results, including the comparison between the two species, are summarized in the first paragraph of the conclusion. While direct comparisons of gene expression levels or hormone concentrations between species are challenging due to the methods used (different reference genes, different plant material), we have focused on comparing the patterns and correlations of ratios of parameters from wild-types versus Ri genotypes across both species. We believe, that this is the most crucial aspect of the manuscript, is methodically sound and enabled us to observe the overall species-de- and -independent impact of wild-strain T-DNA on the phenotype and hormone profiles of Ri genotypes. Additionally, we have elaborated on optimizations for future experimental designs and the broader impact of this study.

Reviewer #2: The paper is a nice combination of different analysis on Ri plants and is valuable for everyone working with the technology. As far as I am aware of, the technology is always described as being a non-GMO technology as long as natural strains are being used. As such, to avoid confusion, I would like to have the paragraph (lines 50-57) removed.

- We sincerely thank the reviewer for their kind words about the value of our study. We agree with the suggestion to avoid confusion regarding the regulatory status. We have removed the specified paragraph and replaced it with a brief remark noting that these plants are generally considered non-GMO when natural strains are used.

Regarding the Gene expression analysis:
- According to the MIQE guidelines, you have to write RT-qPCR instead of qRT-PCR.

- Thank you for pointing this out. We have corrected the terminology to "RT-qPCR" throughout the manuscript to comply with MIQE guidelines.

- have you also included a NTC? And how sure are you about the fact that there is no gDNA contamination? I don't see the use of noRT samples and for bacterial (T-DNA) genes, the development of intron-spanning genes is not possible.

- This is an important point. NTCs were included for all perfomed qPCR runs and can be found in in the supplementary file S1_Raw_Data.xlsx in the sheet “Expression data”. An additional indication was added in the Materials and Methods section of the manuscript to highlight this. The RNA-extraction kit involves digestion of remaining gDNA with DNAseI. After cDNA synthesis, samples were assessed for potential genomic DNA contamination using intron-spanning actin primers. This approach was used to indicate general plant gDNA contamination in the samples assuming that all gDNA had been removed and therefore was not additionally performed for the transgenes. The respective procedure is now included in the manuscript and the actin primer sequences have been added to the supplementary primer list (Supplementary file S3 Materials and Methods, Table B).

Regarding the plant hormone analysis:
samples of 100-200 mg coming from 4-7 replicates were taken. This sounds as a huge variation to me. Could this have had an impact on the outcome?

- We acknowledge the reviewer's observation regarding sample variation. The standard deviations (SDs) are documented in supplementary file S2, sheet "Hormone data". While some variation exists, we applied robust statistical procedures, including log-transformation and residual analysis, to ensure the validity of our linear models. Despite the variation, we were able to identify significant differences and consistent patterns in the hormone heatmap and the correlation analyses. We have discussed in the conclusion that future experimental designs could be optimized to further increase statistical power, but we are confident that the current results support our conclusions.

Line 506 - typo: correlation auf... should be of

- We have corrected this typo, thank you!

Reviewer #3: This is a valuable manuscript that investigates the effects of rol genes on morphological characteristics and endogenous composition. The manuscript is clearly written and well structured.

- We thank the reviewer for this positive feedback and for taking the time to review our work.

I have only a few minor comments, which are listed below.
-In the caption of Table 1, please also specify the R. rhizogenes strain that was used.

- We thank the reviewer for this helpful suggestion. We have added the strain information to the caption of Table 1.

-If possible, please include additional information on the copy number of the introduced genes, as gene copy number can influence plant characteristics.

- We agree that correlating copy number with gene expression would be highly interesting. Unfortunately, establishing a reliable protocol for copy number identification via droplet digital PCR (ddPCR) has proven to be surprisingly difficult for these genotypes. As we are still working on optimizing this method, it was not possible to include this data in the current study.

-Several data sheets are mentioned in the manuscript; please ensure that all such references consistently refer to the appropriate supplementary data sheets (include S number).

- We have double-checked all references to supplementary data. We have ensured that every mention of a data sheet is now accompanied by the corresponding Supplementary File number (e.g., S1, S2) to avoid confusion.

Typographical corrections:
• Line 506: “auf” should be corrected to “of”.

• Line 559: “Since” should not be capitalized.

- We have corrected both typographical errors, thank you!

Reviewer #4: Dear authors,

The article's topic is very interesting. However, the work itself is very crude, both in its presentation and in the presentation of the most significant data.

- We thank the reviewer for finding the topic interesting and for their critical assessment. We have carefully addressed the specific concerns raised to improve the presentation and clarity of our data.

1. In the introduction, the influence of auxins on the expression of rol genes was ignored, although it is an important factor in the mutual regulation of their expression. The work was stated to be devoted to the study of hormone homeostasis, but the results do not confirm this.

- We thank the reviewer for highlighting this important interaction. We have added this information to the dedicated paragraph in the Discussion section detailing how auxins can influence rol gene expression. We also wish to clarify that the primary focus of this study was to observe the impact of the whole, wild-strain T-DNA on several key hormones across two distinct species, rather than to uncover specific regulatory mechanisms. This has not been done so far with the whole, wild-strain T-DNA. Most studies investigated only single rol genes and also single species, sometimes not even in plants (hairy root cultures, protoplasts). We believe this species-independent perspective provides valuable information for breeders and researchers working with Ri genotypes.

2. In the section on expression in the Materials and Methods chapter, it's unclear whether melting curve analysis was used for each sample or only for primer testing. This is an important consideration when analyzing transgenes.

- We apologize if this was unclear. We now have clarified in the Materials and Methods section that melting curves were generated and assessed for all reactions—both during primer testing and during the actual expression analysis—to ensure specificity. We also added information regarding the validation of amplicons via agarose gel electrophoresis.

3. The most important stages of hormone analysis are included in the appendix. The instrument used should be included in the body of the article.

- We agree that the analytical instrumentation should be clearly stated in the main text. Therefore, we have added the details on the LC–MS/MS system to the Materials and Methods section.

Why was such a complex, multi-step sample preparation method used? This type of sample preparation is difficult to standardize.

- The multi-step sample preparation, including SPE clean-up, was deliberately selected for two major reasons: (i) removal of matrix constituents and potential interferents, which is crucial for LC–MS/MS quantification of phytohormones in complex plant extracts; and (ii) concentration of analytes, enabling reliable detection and quantification of low-abundance hormones.
  While the Reviewer notes that such sample preparation can be difficult to standardize, in practice SPE-based workflows are widely used and well-established in phytohormone analysis. The applied procedure follows standard, reproducible steps (fixed sorbent type, solvent composition, volumes, washing/elution sequence), and its main goal is to ensure reduced matrix effects and improved analytical performance.
  There are hundreds of primary and secondary metabolites present in the extracts at much higher concentrations than phytohormones. These substances cause ion suppression in LC-MS/MS analysis, reducing analyte signal intensity (sensitivity), particularly in electrospray ionization (ESI).
- To address potential concerns regarding quantitative accuracy when using multi-step sample preparation, we emphasize that isotope-labelled internal standards were added at an early stage of extraction. This approach is standard in quantitative LC–MS/MS and compensates for potential losses during extraction, SPE, evaporation and reconstitution steps, as well as for matrix effects in ESI. Therefore, the applied internal standard strategy effectively minimizes any negative impact of complex sample preparation on quantification quality and comparability between samples.

Furthermore, the detection limit of hormones must be specified in ng, as calculated for the samples, not in Fmol.

- We agree that detection limits should be interpretable in the context of sample material. We reported LOD/LOQ in fmol, which is a common unit in LC–MS/MS quantitative analysis and directly reflects the instrument’s absolute sensitivity. However, to align with the Reviewer’s suggestion, we have converted the values to express detection limits as mass equivalent (ng) per sample, based on the actual sample mass/extraction volume used. This will make the analytical performance easier to interpret.

The device used does not have sufficient resolution to detect differences in hormonal profiles. Multi-step sample preparation allows for the concentration of hormones for detection, but may impact the accuracy of quantitative analysis.

- We respectfully clarify that the applied method relies on triple quadrupole LC–MS/MS in MRM mode, which is specifically designed for high selectivity and sensitivity in targeted quantification. In this type of analysis, the key determinant is not chromatographic “high resolution” in the sense of HRMS, but rather: chromatographic separation sufficient to avoid co-elution, and the selectivity of MRM transitions combined with retention time matching and internal standard correction.
  The LC–MS/MS platforms used here has been successfully and extensively applied to targeted profiling of multiple phytohormone classes in numerous published studies. Thus, the instrumentation is fully adequate for the analytical task undertaken in this work.
- We agree that very low analyte concentrations increase relative noise and may reduce the statistical power to detect differences between genotypes. This means, that impacts on accuracy might result in enhanced standard deviations. Reviewer 2 has raised a similar concern, which we have answered as follows:
  “We acknowledge the reviewer's observation regarding sample variation. The standard deviations (SDs) are documented in supplementary file S2, sheet "Hormone data". While some variation exists, we applied robust statistical procedures, including log-transformation and residual analysis, to ensure the validity of our linear models. Despite the variation, we were able to identify significant differences and consistent patterns in the hormone heatmap and the correlation analyses. We have discussed in the conclusion that future experimental designs could be optimized to further increase statistical power, but we are confident that the current results support our conclusions.”

4. In this work, IAA is of greatest interest because it is involved in the mutual regulation of the expression of rol genes, especially the rolA and rolB genes. Unfortunately, it's impossible to establish relationships between IAA content and rol gene expression in roots and shoots in this study. Material of varying ages and origins was used. Am I correct in my understanding?

- You are absolutely correct in your understanding, and we appreciate you highlighting the critical role of IAA, particularly regarding its interplay with rolA and rolB. We share your view that this hormone is of special significance in the context of *Rhizobium rhizogenes*-mediated transformation.
- Regarding your observation about the plant material: You are correct that samples for morphological parameters, gene expression, and hormone profiles were collected at different timepoints and, in some instances, analyzed in different laboratories. Consequently, we strictly avoided making direct quantitative comparisons between different organs or comparing absolute concentrations between different hormones.
- However, we would like to emphasize that the primary focus of this study was not to investigate the specific causal regulatory mechanisms of single genes, or to compare concentrations between organs, but rather to uncover the broader correlations and patterns induced by the integration of the whole wild-strain T-DNA. While the experimental design does not allow for a cause-and-effect analysis between IAA and gene expression across organs, it allows for a very robust comparison between the Wild-Type and the Ri genotypes within each parameter. This comparison—identifying how the Ri phenotype deviates from the wild-type in terms of morphology, expression, and hormonal profile—was the most relevant aspect of our study.
- We agree that establishing a relationship between IAA and rol expression is of interest. We have addressed this by performing correlation analyses of Ri/wild-type ratios within the context of the whole wild-strain T-DNA effect. Furthermore, to account for the importance of auxin, we have created a specific paragraph in the Discussion section. There, we have integrated findings from existing literature regarding the interaction of single T-DNA genes and auxins in various systems to interpret our results and suggest which auxin-rol gene interactions might be at play.
- While our study is descriptive by nature and cannot prove or disprove a specific regulatory mechanism, we believe its unique strength lies in the comparative analysis of two phylogenetically (clade rosids vs. asterids) and physiologically (woody vs. herbaceous) distinct species. This approach allows us to observe the "net effect" of wild-strain T-DNA integration, providing valuable insights into species-dependent and independent patterns that are highly relevant for breeders and the broader scientific community.

5. Gene expression analysis is only provided in the supplement. It's very difficult to understand what can be compared with what. Is it possible to compare expression in shoots and roots? Or are they also of different ages and origins? Is it possible to compare the expression of the rola, b, and c genes within a single sample?

- We understand the reviewer's concern. To make it easier for the reader to understand the experimental design, we have created a table for each procedure, which can be seen in the supplementary file “S3 Materials and Methods” in Table A “Overview of all experiments/batches”.
- As noted in the results section, graphical comparisons across species were deemed inadvisable because different reference genes were required for rose and potato, making relative expression levels incomparable. Additionally, we could not calculate ratios against the wild-type for gene expression as the wild-type does not contain these genes. However, the ANOVA results (supplementary file S4) demonstrate that rol gene expression levels differed significantly between Ri genotypes, which is a prerequisite for the most important subsequent correlation analysis and PCA.

6. It seemed that the authors were not confident in this data or for some reason could not analyze it and determine the patterns.

- We assure the reviewer that we are confident in our methods, data and the patterns we have identified. All these results we have summarized in the first paragraph of the Conclusion, and we believe, that the statistical methods, we applied, were sound. At the end in the discussion section, we discuss possibilities for improvement of the current study design to increase statistical power. But this does not devalue the results we achieved. Some comparisons are not possible, which might be possible with a different experimental design, but, as described, these comparisons are of lesser interest, compared to the comparison of wild-type to Ri genotype.

7. Since the authors draw conclusions about IAA and JA (Table 2), it is necessary to place these two hormones in a histogram separate from the heatmap. Since the authors draw conclusions about expression (Table 2), it is necessary to display these data in the histogram in the results.

- Following the reviewer’s suggestion, we have converted the means and standard deviations from the supplementary file S2 “Data Summary” for morphology, hormones, and expression into graphical formats. For readers, who are not satisfied with the hormone heatmap and want to have a closer look into the integrity of the raw data, the graphs are now available in the supplementary file S4 “Results”.
- We have chosen to retain the heatmap in the main text as it allows for a more efficient visual comparison of the relative changes (fold-changes) across multiple genotypes and parameters, which is the central focus of our comparative study. We also retained the spreadsheet for display of precise values and additional ratios to the wild-type.
- Regarding showing of expression data, we have discussed, why this can be misleading (no wild-type, different reference genes for rose and potato), and we therefore strongly suggest to retain it only in the supplementary files. In addition, this does not provide any further value for explaining the correlation analysis, as the correlation was not done on the raw data, but on scaled expression values, due to the missing wild-type. To make this clear, this is pointed out in the Materials and Methods section, the Results section and the table heading.

8. I was very surprised that the discussion focused on the effects of natural transformants, while the work was about direct transgenesis. And if data on rol gene expression and hormone levels were obtained, they need to be discussed in light of similar publications.

- We apologize for any confusion. To clarify, the genotypes in this study were created via *Rhizobium*-mediated transformation using the wild-type *R. rhizogenes* strain ATCC 15834. This makes them "artificial natural transformants" containing the complete wild-type T-DNA, rather than constructs with single genes. This is a key novelty of our study, distinguishing it from research on single rol genes. We have discussed our results in the context of single-gene studies because they represent the bulk of the available literature. We agree, that whole, wild-strain T-DNA can be expected to have a different impact on hormonal profiles, compared to single T-DNA genes, and that the discussion with the available literature is therefore difficult.

advisory comments
9. If the authors doubt the obtained data (hormone levels and rol gene expression) and have difficulty interpreting them, I would suggest retaining only the morphometric data. The hormone levels and rol gene expression should be refined. The expression analysis should be conducted more thoroughly and on a homogeneous sample (age and origin).

- As stated previously, we do not doubt the validity of our hormone or gene expression data. We believe the dataset is robust enough to support the conclusions drawn regarding the phenotypic and hormonal shifts in Ri genotypes. We have identified limitations, but they do not invalidate the significant novel findings and patterns we have shown.

10. It would be very interesting if IAA content and expression were measured in samples of the same age to identify patterns. IAA is most effectively measured using ELISA. Using diethyl ether-based concentration, sample preparation is simpler than that used in this study. And the method is significantly more sensitive. I don't insist on this for this job, but it might be interesting in the future.

- We thank the Reviewer for this interesting suggestion. However, in the present study we intentionally used targeted LC–MS/MS (triple quadrupole, MRM) with isotope-labelled internal standards, as this approach is currently considered one of the most robust methods for quantitative phytohormone profiling in complex plant matrices. Importantly, our aim was not limited to IAA quantification only, but included a broader hormonal panel comprising IAA, several cytokinins, and stress-related hormones. Therefore, the applied workflow was selected to provide a unified, selective and quantitatively reliable analytical platform for multiple hormone classes within the same samples. LC–MS/MS ensures high selectivity and reliable quantification by compensating for matrix effects and analyte losses during sample preparation. In contrast, immunoassays such as ELISA may suffer from cross-reactivity with structurally related compounds and are generally less comparable across laboratories.
  Regarding diethyl ether-based concentration, although such a workflow may appear simpler, plant extracts contain numerous matrix constituents that can interfere with analysis; therefore, SPE clean-up and concentration were applied to reduce interferents and improve analytical performance and data reliability. Measuring IAA levels together with gene expression in samples of identical developmental stage is indeed an interesting perspective for future work; however, such additional experiments are beyond the scope of the present manuscript.

11. It's possible that if the data provided is incorrect, data conflicts may arise in your or others' future research. Since this topic is very interesting and has real potential for practical application, I strongly recommend not rushing into publishing data if the authors themselves are not satisfied with it.

- We have carefully reviewed our data and methods and remain confident in the validity of our results. Our analysis focused on the comparison of wild-type vs. Ri genotypes, a comparison in which we have high confidence. Nevertheless, we have explicitly stated the limitations of the study design and took care to ensure that the conclusions drawn from the valid data are formulated with appropriate caution.

Minor comments:
• Carefully check the formatting of the article in accordance with the requirements of the journal. In addition, the work requires language and professional editing.

- We have adjusted all formatting, headings, and file naming to meet the journal's requirements. The manuscript has now been professionally edited by the "American Journal Experts" to ensure high language quality.

• When mentioning a plant in the title, abstract, keywords, and at the first mention in the introduction, the full Latin name must be given (with author). When first mentioned in an introduction, the family must be indicated.

- We have corrected the Latin names and included family indications at the first mention.
